# The Impact of Portal Vein Thrombosis on the Prognosis of Patients With Cirrhosis: A Retrospective Propensity-Score Matched Study

**DOI:** 10.3389/fmed.2021.685944

**Published:** 2021-06-28

**Authors:** Zhiji Chen, Tao Ran, Haiyan Cao, Feng Xu, Zhi-hang Zhou, Song He

**Affiliations:** Department of Gastroenterology, Second Affiliated Hospital of Chongqing Medical University, Chongqing, China

**Keywords:** cirrhosis, survival, decompensation, portal vein thrombosis, propensity score matching

## Abstract

**Objectives:** To investigate the impact of portal vein thrombosis (PVT) on cirrhosis decompensation and survival of cirrhosis.

**Methods:** In this retrospective observational study between January 2012 and August 2020, 117 patients with cirrhotic PVT and 125 patients with cirrhosis were included. Propensity score matching (PSM) was applied to reduce the bias. The clinical characteristics of non-tumoral PVT in cirrhosis and its influence on cirrhosis decompensation and survival were analyzed.

**Results:** The median follow-up for the PVT group was 15 (8.0–23.0) months and for the non-thrombosis group 14 (8.0–23.5) months. The presence of PVT was related with esophageal varices, higher Child-Pugh score and MELD score (*P* < 0.05). Most PVTs were partial (106/117). Non-occlusive PVT disappeared on later examinations in 32/106 patients (30.19%), of which six patients reappeared. All the 11 patients with occlusive PVT remained occlusive, among which five patients (45.45%) developed portal cavernoma. There was no significant correlation between PVT and decompensation or survival before or after PSM. Multivariate analysis identified only Child-Pugh score (HR = 2.210, 95% CI: 1.332–3.667) and serum sodium level (HR = 0.818, 95% CI: 0.717–0.933) as independent factors for death.

**Conclusion:** Though PVT is associated with greater Child-Pugh score and MELD score, it has no significant impact on the progression of cirrhosis.

## Introduction

Portal vein thrombosis (PVT) is a common complication of patients with cirrhosis. It is associated with relative venous stasis caused by portal hypertension, endothelial injury, hypercoagulability, splenectomy and other factors. Acute PVT can have severe abdominal pain, while chronic PVT can be asymptomatic. Many asymptomatic PVT have been accidentally discovered by the widespread application of medical imaging technology. It was reported that the 5-year cumulative incidence of PVT was 10.7% after regular follow-up of 1,243 Child A and B cirrhosis (1). Non-tumoral PVT is present at liver transplantation in 5–26% of cirrhotic patients (2). Based on current data, the annual incidence of PVT in patients with advanced cirrhosis may be 10–15% (3). The incidence of PVT is significantly higher in decompensated cirrhosis (10–25%) than in compensated cirrhosis (1–5%) (4). Although it was estimated from clinical experience that non-tumoral PVT increases the incidence of refractory ascites, worsens liver function, and ultimately reduces the survival rate of patients, the conclusions from clinical studies are controversial, largely due to the bias in baseline features. Propensity score matching (PSM) is a good way to reduce selection bias.

We retrospectively explored the impact of PVT on the hepatic decompensation and survival rate in 117 patients with cirrhotic PVT and 125 patients without PVT. We found that the Child-Pugh score and MELD score of the PVT group were higher than those of the non-thrombosis group (*P* < 0.05). PVT was mostly partial and the most common clinical outcome was unchanged or improvement. However, there was no significant correlation between PVT and decompensation or survival before or after PSM. All together, though PVT is associated with greater Child-Pugh score and MELD score, it has no impact on the progression of cirrhosis.

## Materials and Methods

### Patients and Study Design

This study selected patients who were hospitalized for cirrhosis in the Second Affiliated Hospital of Chongqing Medical University from 2012 to 2020. Inclusion criteria included: Age >18 and <80 years, clinical diagnosis of cirrhosis (presence of irregular margins on ultrasound, portal hypertension with laboratory evidence of chronic liver disease) (5). The exclusion criteria were as follows: patients with malignant diseases (including history of hepatocellular carcinoma); patients who received anticoagulant treatment during follow-up; prior transjugular intrahepatic portosystemic shunt (TIPS) or surgical shunt; (6) patients with history of bleeding or blood products (red blood cells, platelets, plasma) transfusion in the past 2 weeks. This study was approved by the institutional ethics committee of the Second Affiliated Hospital of Chongqing Medical University.

### Portal Vein Thrombosis Diagnosis

When abdominal ultrasound found solid endoluminal material in the trunk and branches of the portal vein, it was suspected that there was a portal vein thrombosis. Patients with suspected PVT underwent abdominal computed tomography (CT) or magnetic resonance (MRI) to confirm the diagnosis. Occlusive PVT was defined as no flow visible in portal vein lumen on imaging or Doppler study (7). Otherwise PVT was considered non-occlusive. The natural course of thrombosis was observed in our study, which was classified into three categories based on the changes in the degree or extension seen on the imaging: improved or worsened appearance for 50% change or more and unchanged appearance for less than that (6).

### Follow-UP

All patients performed imaging and laboratory tests at least every 6–12 months. Primary endpoint: all-cause death during follow-up; secondary endpoint: decompensation (refractory ascites, hepatic encephalopathy, variceal bleeding, jaundice, or serum bilirubin >45 mol/L) (1). Esophageal varices were graded according to the Paquet's classification (8). The management of complications of cirrhosis was carried out according to current international guidelines (9–12).

### Statistical Analysis

Kolmogorov-Smirnov test was used to test the normality of continuous variables. Normally distributed variables were compared with Student's *T*-tests, expressed as mean ± standard deviation (SD). Non-normally distributed variables were compared with the Mann-Whitney *U*-test, expressed as the medians with interquartile ranges (IQRs). Categorical variables were compared with the χ^2^ or Fisher's exact tests, expressed as counts and percentages. Cox proportional hazards regression model with forward stepwise elimination was used to determine risk factors for decompensations and survival. Ninety-five percent confidence intervals (95% CI) were computed. Multivariate models included variables significantly associated with the outcome in univariate analyses at a level of 0.1.

To reduce the probability of selection bias, propensity score matching (PSM) was performed. Propensity scores were estimated using based on serum albumin level, hemoglobin level, Child-Pugh score, MELD score, the history of splenectomy, varices grade III/IV according to Paquet, portal vein diameter and D-dimer. Patients in the PVT group were matched to those in the non-thrombosis group (1:1), with the nearest neighbor estimated propensity score within a range of 0.02 standard deviation.

Data analysis used IBM SPSS Statistics version 25.0. *P* < 0.05 was considered as statistically significant.

## Results

### Patient Baseline Characteristics

Initially, 1,187 patients with cirrhosis were evaluated, of which 945 were excluded (221 with malignant diseases, 112 with insufficient laboratory data, 86 with anticoagulant treatment during follow-up, 74 with transjugular intrahepatic portosystemic shunt or surgical shunt, 323 with history of bleeding or blood products transfusion in the past 2 weeks, 89 with inadequate follow-up duration, 40 with patients with hepatic encephalopathy, refractory ascites, and recent variceal bleeding in the baseline). Finally, 117 patients with cirrhotic PVT and 125 patients with cirrhosis who were hospitalized during the same period were enrolled. All patients with PVT did not receive anticoagulation and TIPS treatment before or during the follow-up period. No significant difference was observed between the two group in sex, age, or cirrhosis etiology. The serum albumin and hemoglobin level of the PVT group were significantly lower than those of the non-thrombosis group (*P* < 0.05), and the Child-Pugh score and MELD score were higher than those of the non-thrombosis group (*P* < 0.05). There were also differences in the history of splenectomy, varices grade III/IV according to Paquet, portal vein diameter, and D-dimer between the two groups (*P* < 0.05). Detailed patient characteristics are presented in Supplementary Table 1.

### Characteristics and Natural Course of PVT

Among the 117 PVT patients, 62 patients (52.99%) had thrombosis involving the trunk and branches of the portal vein, and only 11 patients (9.40%) had occlusive PVT. During the follow up period, 44.44% of PVT extended to the splenic vein or superior mesenteric vein (Table 1). Non-occlusive PVT disappeared on later examinations in 32/106 patients (30.19%), of which six patients reappeared. Totally, 11/117 (9.40%) patients with PVT had progression of the thrombosis (Supplementary Table 2). All the 11 patients with occlusive PVT remained occlusive, but 5/11 patients with occlusive PVT (45.45%) developed portal cavernoma.

**Table 1:** Characteristics of PVT in patients with cirrhosis.

**Patients with PVT (*****n*** **=** **117)**	
**Site of PVT**, ***n*** **(%)**	
Only trunk	32 (27.35%)
Only branch	23 (19.66%)
Trunk and branches	62 (52.99%)
**Degree of PVT**, ***n*** **(%)**	
Occlusive	11 (9.40%)
Non-occlusive	106 (90.60%)
**Extension of PV system occlusion**, ***n*** **(%)**	
PV alone	65 (55.56%)
Extension into SV	4 (3.42%)
Extension into MV	36 (30.77%)
Extension into SV and MV	12 (10.25%)

*PVT, portal vein thrombosis; PV, portal vein; SV, splenic vein; MV, mesenteric Vein*.

### Clinical Outcomes

The median follow-up for the PVT group was 15 (8.0–23.0) months and for the on-thrombosis group 14 (8.0–23.5) months. There was no significant difference in the incidence of refractory ascites, hepatic encephalopathy, variceal bleeding, and decompensation between the two groups (*P* > 0.05) (Supplementary Table 3). Occlusive PVT also had no significant effect on decompensation (χ^2^ = 0.031, *P* = 0.861). Factors associated with decompensation of cirrhosis by Cox univariate regression analysis are shown in Supplementary Table 4. Multivariate COX regression analysis found that the independent influencing factors of decompensation in patients with cirrhosis were esophageal varices (HR = 3.187, 95% CI: 1.601–6.343, *P* = 0.001), endoscopic treatment (HR = 0.834, 95% CI: 0.706–0.984, *P* = 0.032), serum sodium level (HR = 0.903, 95% CI: 0.853–0.955, *P* < 0.001) and spontaneous portosystemic shunts (HR = 2.338, 95% CI: 1.314–4.162, *P* = 0.004) (Table 2). No relationship has been observed between PVT and decompensation in different Child-Pugh class and MELD score.

**Table 2:** Multivariate analysis to determine predictive factors for decompensation and death.

	***P*-values**	**HR**	**95% CI**
**Decompensation**			
Esophageal varices (Paquet's grade III/IV)	0.001	3.187	1.601–6.343
SPSS	0.004	2.338	1.314–4.162
Endoscopic treatment	0.032	0.834	0.706–0.984
Serum sodium level	<0.001	0.903	0.853–0.955
**Death**			
Child-Pugh score	0.002	2.210	1.332–3.667
Serum sodium level	0.003	0.818	0.717–0.933

*SPSS, Spontaneous portosystemic shunts*.

Overall, 10/242 (4.13%) patients died, among which five are associated with multiple organ failure and the other five with gastrointestinal bleeding. Factors associated with death by univariate Cox regression analysis are shown in Supplementary Table 4. There was no influence of PVT on survival. Multivariate analysis identified only Child-Pugh score (HR = 2.210, 95% CI: 1.332–3.667, *P* = 0.002) and serum sodium level (HR = 0.818, 95% CI: 0.717–0.933, *P* = 0.003) as independent factors (Table 2).

### Propensity-Matched Cohort

**Figure 1:**
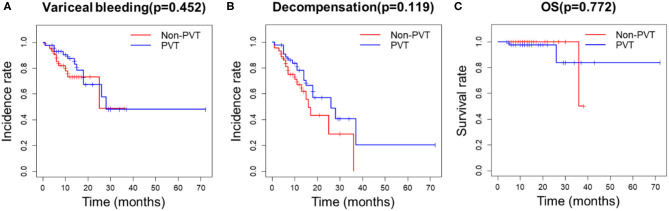
Kaplan-Meier curves for PVT and non-PVT groups. **(A)** PVT had no effect on variceal bleeding, **(B)** decompensation of cirrhosis and **(C)** survival after PSM.

After propensity score matching, 44 patients remained in each group (Table 3). There was no significant difference in the baseline features between the two groups. We found that PVT had no effect on variceal bleeding (Figure 1A) and decompensation of cirrhosis (Figure 1B). No significant difference in survival time was found between the two groups (Figure 1C). Meanwhile, esophageal varices (HR = 4.428, 95% CI: 1.633–12.008, *P* = 0.003), serum sodium level (HR = 0.921, 95% CI: 0.862–0.984, *P* = 0.015) and spontaneous portosystemic shunts (HR = 3.062, 95% CI: 1.363–6.880, *P* = 0.007) independently predicted cirrhosis decompensations (Table 4). Because of the small number of deaths, we did not find independent risk factors for death after PSM.

**Table 3:** Propensity-matched study cohort.

	**With PVT (*n* = 44)**	**Without PVT (*n* = 44)**	***P*-values**
Age (years)	54.0 ± 11.4	54.6 ± 11.7	0.775
Male gender	30 (68.18%)	26 (59.09%)	0.375
Etiology of cirrhosis (HBV/alcohol/other)	29/5/10	28/5/11	0.968
Child-Pugh score	7.0 (6.0–8.0)	7.0 (6.0–8.0)	0.594
MELD score	9.3 (8.5–11.8)	8.8 (8.1–10.7)	0.367
Serum albumin (g/L)	35.5 ± 5.1	35.1 ± 6.3	0.785
Hemoglobin (g/L)	99.4 ± 24.3	99.8 ± 25.5	0.935
Portal vein diameter (mm)	16.0 (15.0–17.2)	16.0 (13.3–18.4)	0.970
D-dimer (μg/L)	0.4 (0.1–0.6)	0.2 (0.1–0.7)	0.468
History of splenectomy	2 (4.55%)	2 (4.55%)	1.000
Diabetes	8 (18.18%)	7 (15.91%)	0.777
Esophageal varices (Paquet's grade III/IV)	32 (72.73%)	33 (75.00%)	0.808
SPSS	8 (18.18%)	9 (20.45%)	0.787

*PVT, portal vein thrombosis; HBV, hepatitis B virus; MELD, Model for End-Stage Liver Disease; SPSS, Spontaneous portosystemic shunts*.

**Table 4:** Multivariate analysis to determine predictive factors for decompensation and death (propensity-matched study cohort).

	***P*-values**	**HR**	**95% CI**
**Decompensation**			
Esophageal varices (Paquet's grade III/IV)	0.003	4.428	1.633–12.008
SPSS	0.007	3.062	1.363–6.880
Serum sodium level	0.015	0.921	0.862–0.984
**Death**			
Child-Pugh score	0.085	1.996	0.909–4.380

*SPSS, Spontaneous portosystemic shunts*.

## Discussion

PVT is a common complication in patients with cirrhosis, and its pathogenesis can be explained by the Virchow's triad (blood flow stasis caused by portal hypertension, vascular endothelial damage and blood hypercoagulability). With the progress of liver disease, the flow of the portal vein decreases and ectopic intestinal bacteria increase, which lead to vascular endothelial damage (13). In addition, elevated endothelial-derived factor VWF and decreased protein C resulted in a relatively hypercoagulable state (14). As a result, the incidence of PVT is higher in patients with more advanced cirrhosis. Nery et al. followed up 1,243 patients with cirrhosis for 47 months and found that the independent risk factors for development of PVT were baseline esophageal varices and prothrombin time, but not with prothrombotic mutations (1). Consistently, our study also found that patients with PVT have worse liver function and a higher proportion of severe esophageal varices.

There are few studies on the natural course of PVT. A retrospective study found that thrombosis was improved in 47.60 %, unchanged in 45.20 %, and worsened in 7.20% (6). Our data showed that 90.59% of PVT were partial thrombosis, 27.35% of PVT disappeared completely during follow-up, and only 9.40% of PVT had progressed significantly. It can be seen that PVT is mostly partial thrombosis and the most common clinical outcome is unchanged or improvement. Qi X et al. proposed risk stratification for PVT: transient PVT was defined if a thrombus within the portal vein spontaneously disappears within 3 months in the absence of antithrombotic treatment (15). But so far, no predictor of transient PVT has been found. All the 11 patients with occlusive PVT remained occlusive, but 5/11 patients with occlusive PVT (45.45%) developed portal cavernoma. Portal cavernoma is considered to be one of the characteristics of chronic PVT and can maintain blood supply to the liver.

The effect of PVT on the decompensation or survival is controversial. Some studies did report that PVT was associated with increased mortality (16–18). But other studies showed that partial thrombosis is common in the clinic and there is no significant association (1, 6, 19, 20). Nery et al. (1) adjusted for baseline liver function and found that the formation of PVT did not increase the risk of decompensation in patients with cirrhosis. The independent risk factors for decompensation were esophageal varices (≥grade2) and prothrombin time. A recent prospective study (19) followed up 241 patients with cirrhosis for 29 months and found that PVT development did not independently predict cirrhosis decompensations or lower OLT-free survival. This can be at least partially caused by the fact that some of these studies did not appropriately adjust for differences in baseline liver function between PVT and non-thrombosis patients. Because PVT is more likely to occur in advanced cirrhosis, differences in liver function have a greater impact on decompensation and mortality (21). We used propensity score matching to adjust for confounding factors and found that the independent risk factors for decompensation were esophageal varices, serum sodium level and spontaneous portosystemic shunts. We consider that PVT has little effect on liver blood flow as most of the PVT is partial and occlusive thrombosis can form collateral vessels, which will reduce the portal vein tension. PVT may be a marker of liver disease decompensation, rather than a direct cause of portal hypertension and liver disease decompensation. Large prospective studies are needed to reveal the effect of PVT on the outcome of cirrhosis patients.

A systematic review analyzed 25,753 liver transplant patients and found that only patients with occlusive thrombosis had a reduced survival rate after transplantation (22). Qi X et al. called occlusive PVT or PVT with extensive superior mesenteric vein thrombosis as clinically significant PVT (23) and believe that when clinically PVT is present, the prognosis of cirrhosis will be significantly compromised and anticoagulation therapy will benefit in such patients. However, our data showed that the occlusive PVT was 11.1% (5/45) in decompensated liver disease and was 8.3% (6/72) in the compensated group. The difference between two groups is not statistically significant (*P* = 0.861). Due to limited data, whether clinically significant PVT affects the prognosis of cirrhosis needs more data to verify.

Anticoagulation could be considered in selected cases. Acute symptomatic PVT can cause intestinal ischemia, anticoagulation therapy is recommended. The presence of severe PVT increases the complexity of the operation, reduces the blood supply of the transplanted organ, and decrease survival after transplantation (22). Therefore, the current guidelines generally recommend anticoagulation therapy for patients with PVT who are liver transplantation candidates (9, 24). With newly diagnosed PVT, comprehensive consideration should be given to extent of the thrombosis, presence or absence of attributable symptoms and risk of bleeding (24). When PVT progresses significantly and extends to the superior mesenteric vein, anticoagulation therapy should be considered. If no treatment, regular follow-up is required, and a considerable part of non-occlusive PVT will disappear.

Limitations of our study are mainly related to its retrospective nature. Most of the patients we included were Child-Pugh class A and B, the number of deaths during the observation period was small. Therefore, exploring the independent risk factors of death in cirrhosis has limited significance. Despite these limitations, our study has several strengths. This is a large retrospective study to observe the course of PVT under natural conditions. PSM analysis was applied to control selection bias.

In conclusion, the incidence of PVT is higher in patients with more advanced cirrhosis. The development of PVT cannot independently predict clinical outcome.

## Data Availability Statement

The original contributions presented in the study are included in the article/Supplementary Materials, further inquiries can be directed to the corresponding authors.

## Ethics Statement

The studies involving human participants were reviewed and approved by the Institutional Ethics Committee of the Second Affiliated Hospital of Chongqing Medical University. Written informed consent for participation was not required for this study in accordance with the national legislation and the institutional requirements.

## Author Contributions

ZC, Z-hZ, and SH: conception and design. ZC, HC, and FX: collection and assembly of data. ZC, TR, and HC: analysis and interpretation of the data. ZC and TR drafting of the manuscript. Z-hZ and SH supervised the study and revised the manuscript. All authors contributed to the article and approved the submitted version.

## Conflict of Interest

The authors declare that the research was conducted in the absence of any commercial or financial relationships that could be construed as a potential conflict of interest.
