# Supplementary material for: The Impact of Portal Vein Thrombosis on the Prognosis of Patients With Cirrhosis: A Retrospective Propensity-Score Matched Study
Source: Front Med (Lausanne). 2021 Jun 28;8:685944. doi: 10.3389/fmed.2021.685944 (PMC8275025; doi:10.3389/fmed.2021.685944)
Supplement: Supplementary file 1 [file Table_1.DOCX]

| **TableS1.Patients Characteristics at baseline** | | | |
| --- | --- | --- | --- |
|  | **With PVT**  **（n=117）** | **Without PVT（n=125）** | **P-values** |
| Age (years) | 55.0±10.9 | 53.2±12.3 | 0.235 |
| Male gender | 78（66.67%） | 81（64.80%） | 0.760 |
| Observation period （months） | 15.0（8.0-23.0） | 14.0（8.0-23.5） | 0.762 |
| Etiology of cirrhosis（HBV/Alcohol/Other） | 81/17/19 | 93/9/23 | 0.182 |
| Child-Pugh class（A/B/C） | 34/64/19 | 60/54/11 | 0.007 |
| Child-Pugh score | 7.0（6.0-9.0） | 7.0（5.0-8.0） | 0.001 |
| MELD score | 10.1（8.5-12.1） | 9.2（8.2-11.0） | 0.010 |
| Serum albumin （g/L） | 33.6±5.2 | 35.5±6.4 | 0.012 |
| Serum bilirubin（μmol/L） | 18.9(11.6-24.9) | 14.9（10.1-23.25） | 0.364 |
| APTT（s） | 40.6(37.7-43.8) | 40.5(38.1-43.2) | 0.447 |
| WBC× 10^9/L | 3.3（2.3-4.3） | 3.3（2.4-4.6） | 0.941 |
| PLT× 10^9/L | 66.0（42.0-146.0） | 62.5（44.0-101.0） | 0.279 |
| Serum sodium（mmol/L） | 139.7（134.6-139.7） | 139.2（136.9-142.3） | 0.765 |
| Serum creatinine（μmol/L） | 62.0（53.4-77.2） | 65.2（54.4-76.0） | 0.634 |
| Hemoglobin（g/L） | 96.7±22.8 | 109.4±26.2 | ＜0.001 |
| Portal vein diameter（mm） | 16.5（15.0-18.2） | 15.0（13.2-17.7） | ＜0.001 |
| D-dimer（μg/L） | 0.5（0.1-1.2） | 0.1（0.1-0.7） | ＜0.001 |
| History of splenectomy | 29（24.79%） | 5（4.00%） | ＜0.001 |
| Diabetes | 18（15.38%） | 31（24.80%） | 0.069 |
| SPSS | 26（22.22%） | 18（14.40%） | 0.115 |
| Esophageal varices（Paquet’s grade III/IV） | 89（76.07%） | 71（56.80%） | 0.002 |

Abbreviations: PVT, portal vein thrombosis; HBV, hepatitis B virus; MELD, Model for End-Stage Liver Disease ; APTT, activated partial thromboplastin time ; WBC, white blood cell; PLT, platelet; SPSS, Spontaneous portosystemic shunts.

| **Table S2. Natural history of portal vein thrombosis** | | | | | |
| --- | --- | --- | --- | --- | --- |
|  | disappeared | Improved | Unchanged | Worsened | Total |
| Nonocclusive | 32 | 15 | 48 | 11 | 106 |
| Occlusive | 0 | 2 | 9 | 0 | 11 |
| Total | 32  （27.35%） | 17  （14.53%） | 57  （48.72%） | 11  （9.40%） | 117（100.00%） |

| **Table S3.Comparison of clinical events during follow-up between the two groups** | | | |
| --- | --- | --- | --- |
|  | **With PVT**  **（n=117）** | **Without PVT（n=125）** | **P-values** |
| Refractory ascites | 15（12.82%） | 14（11.20%） | 0.698 |
| Variceal bleeding | 27（23.08%） | 21（16.80%） | 0.221 |
| Hepatic encephalopathy | 11（9.40%） | 8（6.40%） | 0.386 |
| Decompensation | 45（38.46%） | 41（32.80%） | 0.358 |
| Death | 6（5.13%） | 4（3.20%） | 0.667 |

Decompensation: refractory ascites, hepatic encephalopathy, variceal bleeding, jaundice, or serum bilirubin >45 mol/L.

| **Table S4.Factors associated with decompensation of cirrhosis and death by Cox univariate regression analysis** | | | |
| --- | --- | --- | --- |
|  | **P-values** | **HR** | **95% CI** |
| **Decompensation** |  |  |  |
| Hemoglobin | 0.002 | 0.987 | 0.979-0.995 |
| Serum albumin level | ＜0.001 | 0.926 | 0.894-0.959 |
| Child‐Pugh score | ＜0.001 | 1.301 | 1.162-1.457 |
| MELD score | 0.001 | 1.126 | 1.049-1.208 |
| Serum sodium level | ＜0.001 | 0.912 | 0.872-0.953 |
| Endoscopic treatment | 0.029 | 0.863 | 0.756-0.985 |
| Diabetes | 0.046 | 1.596 | 1.009-2.523 |
| Esophageal varices（Paquet’s grade III/IV） | ＜0.001 | 2.803 | 1.668-4.709 |
| D-dimer | 0.031 | 1.194 | 1.016-1.403 |
| SPSS | 0.027 | 1.768 | 1.067-2.927 |
| **Death** |  |  |  |
| Child‐Pugh score | 0.001 | 2.111 | 1.355-3.290 |
| MELD score | 0.009 | 1.344 | 1.075-1.681 |
| Serum sodium level | 0.001 | 0.815 | 0.723-0.918 |
| Endoscopic treatment | 0.026 | 0.292 | 0.098-0.866 |
